# Acorn Weevil Species Diversity and Host Affinity in the Semi-Humid Evergreen Broad-Leaved Forests of Southwest China

**DOI:** 10.3390/insects16060579

**Published:** 2025-05-30

**Authors:** Shengquan Fang, Shaoji Hu, Biao Zhao, Dengpeng Chen, Chunyan Lan, Xinrong Li, Yongping Li, Mingchun Peng, Zihao Wang, Mingyu Ge, Chongyun Wang

**Affiliations:** 1 School of Ecology and Environmental Science, Yunnan University, Kunming 650500, China; shengquanfang@hotmail.com (S.F.); 18213535564@163.com (B.Z.); chendengpeng@itc.ynu.edu.cn (D.C.); lcy_61208@126.com (C.L.); x571142369@163.com (X.L.); mchpeng@ynu.edu.cn (M.P.); w15675011759@163.com (Z.W.); 17387415438@163.com (M.G.); 2 Institute of Ecology and Geobotany, Yunnan University, Kunming 650500, China; 3 Yuxi Institute of Ecology and Environment Science, Yuxi 653100, China; 4 Institute of International Rivers and Eco-Security, Yunnan University, Kunming 650500, China; shaojihu@hotmail.com; 5 Yunnan Key Laboratory of International Rivers and Transboundary Eco-Security, Yunnan University, Kunming 650500, China; 6 School of Agriculture, Yunnan University, Kunming 650500, China; liyp@ynu.edu.cn

**Keywords:** acorn weevils, species diversity, acorn functional traits, semi-humid evergreen broad-leaved forests

## Abstract

Acorn weevils critically impact forest regeneration in semi-humid evergreen broad-leaved forests (SEBFs) by parasitizing and consuming acorns before dispersal. Despite their ecological significance, research on the species diversity of acorn weevils within SEBFs remains limited. To address this gap, we assessed the species diversity and host affinity of acorn weevils across six dominant oak species at 18 locations. We performed DNA extraction and mitochondrial *COI* gene sequencing on weevil larvae and analyzed acorn functional traits (AFTs) from host acorns. Six acorn weevil species across four genera and two families were identified within the dominant acorns of SEBFs. *Curculio dentipes* showed the lowest host specificity, while *Niphades castanea* and *Cyllorhynchites ursulus* were specialist species. Notably, the species diversity of acorn weevils was significantly lower in *Quercus franchetii* than in others. Acorn volume and three secondary metabolite contents, including total phenols, total flavonoids, and tannins, were the primary AFTs influencing weevil species diversity. This study not only advances our comprehension of acorn weevil species diversity and their ecological interactions with oak hosts, but also provides valuable insights for the ecological management of SEBFs in southwest China.

## Simple Summary

Semi-humid evergreen broad-leaved forests are critical oak forest ecosystems in southwest China, yet the species diversity of acorn weevils and their relationship with host acorns are underexplored within this region. In this study, we identified the diversity of six acorn weevil species and their host plant preferences in the region using field surveys, laboratory observations, and molecular analysis. We found that while all dominant acorns were susceptible to weevil consumption, there were significant differences in weevil species diversity among various host acorns, except for *Quercus franchetii*. Moreover, the acorn volume and secondary metabolite content significantly influenced the species diversity of acorn weevil. These insights are instrumental for advancing seed–insect interaction studies and informing ecological management strategies in similar ecosystems.

## 1. Introduction

Insect parasitism and the consumption of pre-dispersal seeds are major causes of seed loss in many plants, exerting an important selection pressure on plant life histories [1,2]. This interaction between plant seeds and their parasites is crucial for the energy flow (i.e., the transfer of energy from one trophic level to another) and nutrient cycles in ecosystems, as well as for maintaining ecosystem function [3,4]. Understanding pre-dispersal predator–seed interactions can inform strategies to control pest populations. This, in turn, can promote plant regeneration and contribute to effective ecosystem management through targeted pest control measures and habitat restoration efforts [5,6].

Seed-parasitic insects, due to their high species diversity and broad host range, often have a significant impact on plant communities, especially on the survival, reproduction, and regeneration of plant communities that contain rare plants. [7,8]. Increasingly recognized as potential “pests”, these insects pose a threat to germination and seedling establishment in numerous plant species [9]. Their role in community composition and regeneration is pivotal, yet our understanding of their species diversity and host affinity remains limited due to challenges in accurately identifying closely related species, especially in their larval stages [10]. The accurate identification of seed-parasitic insects is crucial for understanding species composition and their interactions with host plants, which is essential for ecological research [11]. Traditional insect identification relies on adult morphological characteristics, as larvae are often indistinguishable, even among well-differentiated species [12,13]. Therefore, to elucidate the species diversity of the larval stages of seed-parasitic insects and to refine the estimation of their host ranges, the employment of DNA barcoding techniques is necessary for unambiguously discriminating among species [14,15]. This is not only crucial for sustaining forest ecosystems, but also vital for formulating restoration, management, and conservation strategies, as their interactions directly influence seed production and plant regeneration, which are fundamental to maintaining forest health [16].

Acorns, the distinctive seeds of oaks, play a key role in shaping the regeneration and succession of oak forests, as well as in maintaining the stability of the oak forest ecosystem [17,18,19]. Acorn functional traits (AFTs), as measurable individual-level characteristics, are crucial for insights into plant biology, conservation, resource utilization, and plant–animal interactions [20,21,22]. For instance, larger acorns with thinner pericarps are more accessible to insect larvae, providing them with ample space and nutrients for development [23,24,25]. Additionally, acorns with lower levels of secondary metabolites, such as tannins and phenols, are less defended and thus more palatable to insects [26]. These characteristics of acorns influence the foraging preferences of insect herbivores and play a crucial role in shaping the interactions between acorns and their insect predators. Acorn weevils (Coleoptera: Curculionoidea), which are considered seed parasitoids, significantly impact this process by preying on acorns before they can disperse and germinate [27]. These weevils, such as species in the genus *Curculio* L. 1758, are known to lay their eggs within acorns, where the larvae develop and consume the seed tissue, often rendering the acorn non-viable for germination [27,28]. These progresses potentially reduce the acorn’s ability to grow into a new oak tree and ultimately affect forest regeneration [28]. In response to predation, acorns have evolved a suite of countermeasures to evade predation. These countermeasures include the production of secondary metabolites such as tannins and phenols, which can deter weevil larvae from feeding [29]. However, despite these defences, the impact of weevil predation remains significant, highlighting the complex interplay between acorn traits and insect behaviour in shaping forest ecosystems [3]. To better understand these complex interactions and their broader ecological implications, we focus our study on the semi-humid evergreen broad-leaved forests (SEBFs) of southwest China, where the unique combination of endemic oak species and frequent weevil activity provides a rich context for exploring these dynamics [30,31]. Despite their ecological significance, the species composition of these acorn weevils and their interactions with acorns within SEBFs have not been systematically studied [31,32,33]. This gap in the knowledge limits our ability to develop effective management strategies for these forests.

To address the research questions regarding the species composition and diversity of acorn weevils in SEBFs, their host range and specificity, and the relationship between AFTs and weevil diversity, we conducted extensive sampling and molecular analysis. Specifically, our goal was to test three hypotheses: (1) multiple species of acorn weevils are present and consume acorns in SEBFs; (2) these weevils show clear differences in their host range and specificity; and (3) AFTs affect the diversity of acorn weevils. Our findings could benefit the understanding of the interactions between host acorns and acorn weevils, which is crucial for forest management and biodiversity conservation in oak forests.

## 2. Materials and Methods

### 2.1. Research Area

**Figure 1:**
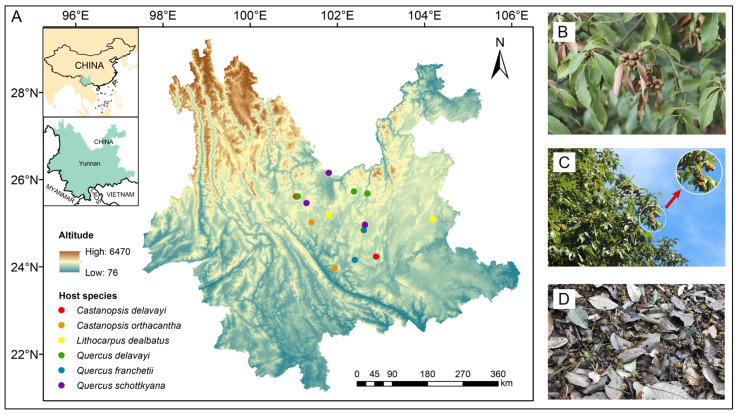
Host acorn sampling site distribution and field conditions in Central Yunnan Plateau. (**A**) Different symbols represent various host acorn sampling sites; (**B**) mature acorns of *Quercus schottkyana*, with some detached from their cupules; (**C**) *Castanopsis orthacantha* acorns enclosed in cupules on branches; and (**D**) *C. delavayi* acorns on the ground, predominantly enclosed in their cupules.

Semi-humid evergreen broad-leaved forests (SEBFs), predominantly located in the Central Yunnan Plateau of southwest China, typically occur in fragmented patches within mountainous areas at elevations between 1500 and 2500 metres (Figure 1) [30]. Similarly to typical humid broad-leaved evergreen forests in East China, these forests are marked by oak species (Fagales: Fagaceae) that are either dominant or co-dominant within the arboreal stratum. However, SEBFs are characterized by the dominance of endemic species such as *Quercus schottkyana* (Rehder and E. H. Wilson, 1916), *Q. delavayi* (Franch., 1899), *Lithocarpus dealbatus* (Rehder, 1919), *Castanopsis orthacantha* (Franch., 1899), and *C. delavayi* (Franch., 1899), with the addition of *Q. franchetii* (Skan, 1899) in the semi-humid and warm regions [34].

### 2.2. Acorn and Weevil Sampling

To identify acorn weevils and their host plants, we sampled host acorns at 18 sites across the Central Yunnan Plateau (23.97–26.16° N, 101.05–104.18° E) during the acorn fall season (when the acorns were mature) in October to November of both 2022 and 2023. The sampling area encompassed the primary distribution area of SEBFs, and the sampled acorns were sourced from six dominant oak species in SEBFs (Figure 1, Table S1). During each sampling session, we randomly selected 3–5 oak mother trees (acorn-producing individuals) per site with a minimum distance of over 10 m between trees, and collected at least 50 mature acorns (including those fallen on to the ground and unshed ones on branches). To obtain more and accurate weevils from the acorns, we screened the collected acorns to ensure that there was no obvious damage or additional infestation. The collected acorns were separately stored in plastic boxes labelled based on site and oak species names. The boxes were tightly sealed to avoid cross-contamination and to prevent the adult insects from escaping after emergence. A total of 5948 acorns were collected for the sampling (Table S1).

All collected acorns were examined daily. We recorded and collected the number and date of weevil larvae and adults that naturally emerged from the acorns, continuing this process until the end of November. Acorns without signs of weevil larvae penetration were dissected, and those containing larvae were subsequently extracted and included in the experimental analysis. We classified all the weevil larvae by the collection sites and the host acorn species, then transferred them to pre-labelled 1.5 mL Eppendorf microcentrifuge tubes with absolute ethanol and stored them at −80 °C until use. A total of 229 larval individuals and four adult weevils were selected for the subsequent molecular work (Table 1), with 20–60 weevil individuals collected from each host plant species. All the collected samples were used for DNA extraction and sequencing.

### 2.3. Molecular Identification

The identification of adult weevils is often straightforward, based on morphological features, but this is not the case for larvae. Therefore, we utilized molecular identification to determine the species of larval weevils found in acorns. We extracted and sequenced DNA from a total of 229 larval weevils and four adult specimens (Table 1). DNA extraction was performed using the High Efficiency Animal Genomic DNA Extraction Kit (TSINGKE) according to the manufacturer’s protocol. After extraction, polymerase chain reactions (PCRs) were conducted with universal primers targeting the mitochondrial DNA gene *COI*: C1-J-2183, 5′-CAACATTTATTTTGATTTTTTGG-3′ and TL2-N-3014, 5′-TCCAATGCACTAATCTGCCATATTA-3′ [35]. The PCR system consisted of a 50 μL volume and was carried out under the following conditions: initial denaturation at 94 °C for 2 min; 5 cycles (denaturation at 94 °C for 30 s, annealing at 50 °C for 40 s, and extension at 72 °C for 1 min); 35 cycles (denaturation at 94 °C for 30 s, annealing at 55 °C for 40 s, and extension at 72 °C for 1 min); and a final extension at 72 °C for 10 min. The amplified products were sequenced in both forward and reverse directions using the Sanger sequencing method on an ABI 3730xl automatic sequencer (Applied Biosystems, Foster City, CA, USA). We compared the obtained sequences with a reference set of adult weevil sequences from China to identify the larval acorn weevils at the species level [2,36].

### 2.4. Measurement of Acorn Functional Traits

Referring to the functional trait classification by Jiménez-Alfaro et al. (2016) [22], we selected 50 sound acorns from different collection sites and species using a quadrat method to measure AFTs, which are categorized into morphological and physiological traits (Table S2). For morphological traits, we assessed five indicators: acorn mass, acorn volume, fruit shape index, pericarp thickness, and cicatrix thickness [37,38]. We measured the acorn mass with an analytical balance (BSA223S, OLABO, Jinan, Shandong, China). Digital vernier callipers (DELL, Ningbo, Zhejiang, China) were used to measure the transverse and longitudinal diameters of the acorns, and the fruit shape index was calculated using the following formula: FSI=LD/TD, where *FSI* is the fruit shape index, *LD* is the acorn’s longitudinal diameter, and TD is the acorn’s transverse diameter. Acorn volume was determined using the water displacement method, utilizing data on the acorns’ diameters (Table S3). After dissecting the pericarp with a scalpel, we measured the pericarp thickness and cicatrix thickness, which are physical defence characteristics, using a thickness gauge (SYNTEK, Huzhou, Zhejiang, China) (Figure S1). For physiological traits, we measured five indicators: water content, starch content (nutrients), and the content of three secondary metabolites (chemical defence substances): total phenols, total flavonoids, and tannins [39]. The water content of acorns was calculated using the following formula: $WC=\frac{{AC}_{F}-{AC}_{D}}{{AC}_{F}}\times 100\%$, where *WC* represents the water content of acorns, *AC_F_* is the fresh weight of acorns, and *AC_D_* is the dry weight of acorns [40]. Starch and the three secondary metabolites (total phenols, total flavonoids, and tannins) were extracted from the acorns using chemical kits from Suzhou Grace Biotechnology Co., Ltd. (Suzhou, Jiangsu, China), and their concentrations were analyzed via enzyme-linked immunosorbent assay (ELISA).

### 2.5. Data Analysis

#### 2.5.1. Weevil Species Identification

We compiled and reviewed the bidirectional sequencing data. If the electropherogram displayed a single, distinct peak, we conducted a Nucleotide BLAST search in the GenBank database to identify matches with published weevil sequences, and downloaded the homologous and outgroup sequences (https://blast.ncbi.nlm.nih.gov/Blast.cgi/; accessed on 12 January 2024) (Table S4) [41]. We aligned all valid sequences and matching sequences in GenBank using the ClustalW programme within MEGA v11.0 (Mega Limited, Auckland, New Zealand) [42,43]. We then assessed the most appropriate nucleotide substitution models for the *COI* gene sequences of the weevils under study, selecting the optimal models based on the BIC and AIC criteria (Table S5) [44]. Based on these assessments, we chose the best-fit model for constructing a maximum likelihood (ML) phylogeny and set the number of bootstrap replicates to 1000. Based on the available phylogenetic relationship studies of Curculionoidea, we selected *Cylas formicarius* (Fabricius, 1793) as an outgroup, searched for *COI* sequences of this species in GenBank and added them to the phylogenetic tree construction [45]. Tree visualization and refinement were performed using MEGA v11.0 and tvBOT web server (https://www.chiplot.online/tvbot.html; accessed on 30 January 2024) [46]. We calculated both intraspecific and interspecific genetic distances using the K2P (Kimura two-parameter) model with 1000 bootstrap replicates in MEGA v11.0.

#### 2.5.2. Host Range and Specificity of the Acorn Weevils

To investigate the host range and specificity of acorn weevils feeding on dominant acorns in SEBFs, we documented the species and abundance of acorn weevils across various acorns, compiling a species-multiplicity data matrix. We employed the “circlize” and “statnet” packages in R 4.3.1 (https://www.r-project.org/; accessed on 12 March 2024) to visualize the correspondence between acorn weevils and their host acorns [47,48]. Additionally, parasitic insects exhibit selective preferences when choosing host plants, often utilizing only parts of the host, a phenomenon known as host specificity [49,50]. However, the count of species and genera that serve as hosts does not adequately measure the host specificity of acorn weevils. Instead, the host specificity score for each weevil species more accurately reflects the breadth of their host range. We determined the host specificity index for the acorn weevils in SEBFs using Hu’s method, represented by the following formula: $S=1/\sqrt{ab^{2}}$, where *S* is the host specificity of the weevil, *a* is the number of the host plant taxa at the species level, and *b* is the number at the genus level [41]. A weevil species parasitizing a single plant species exhibits a host specificity of 1, indicating the highest level of host specificity; conversely, an increase in the number of host plant species correlates with a decrease in host specificity. For the calculations and analyses, we classified weevils with hosts limited to a single genus as specialists, and those with hosts across different genera as generalists, thereby highlighting the variation in host specialization among acorn weevils [51].

#### 2.5.3. Species Diversity of Acorn Weevils

We employed the “iNEXT” package to perform asymptotic diversity estimation based on Hill’s number *q*, using the statistical results from Section 2.5.1 and Section 2.5.2 as the data foundation to compare the species diversity of acorn weevil communities in different host acorns [52]. The model fits three diversity indices: species richness, Shannon–Wiener diversity, and Simpson diversity. Species richness (*q* = 0) focuses only on the presence or absence of species, counting species equally without regard to their relative abundance, with larger values indicating a greater abundance of species in the community. Shannon–Wiener diversity (*q* = 1) is estimated based on the proportional counts of species abundance and can be interpreted as an effective number of common species in the community. Simpson diversity (*q* = 2) is estimated based on dominant species counts to reflect the effective number of dominant species in the community. We estimated the species diversity of acorn weevils using 200 bootstrap resampling iterations with 95% confidence intervals, and the results were presented as rarefaction and extrapolation curves [53,54,55].

#### 2.5.4. Effects of AFTs on the Diversity of Weevils

To determine whether the species diversity of the acorn weevil is significantly correlated with the host’s AFTs, we calculated the mean values of the AFTs (morphology and physiology) of the different hosts and correlated them with the species diversity indices of the acorn weevils (Table S6). We constructed a sample species dataset by treating each host as a sample, where the AFTs of the host act as proxies for the environmental factors within each sample (each row representing one sample and each column representing one environmental factor) [49]. The two datasets were analyzed using direct ordination to explore the relationships between species distributions and environmental factors. Specifically, the proportion of acorn weevil species diversity was explained by the AFTs of the host, and the amount and significance of each trait were explained by acorn weevil species diversity. We employed the “vegan” package in R 4.3.1 to perform detrended correspondence analysis (DCA) on the dataset matrix, and chose canonical correspondence analysis (CCA) to rank the AFTs factors. We applied the *permutest* function to conduct a Monte Carlo permutation test with the results of the CCA analyses, and subsequently used the *envfit* function to assess the significance of each AFT. Thus, we revealed the influence of AFTs of dominant host oaks in SEBFs on the species diversity of acorn weevils.

## 3. Results

### 3.1. Species Composition of the Acorn Weevil in SEBFs

**Figure 2:**
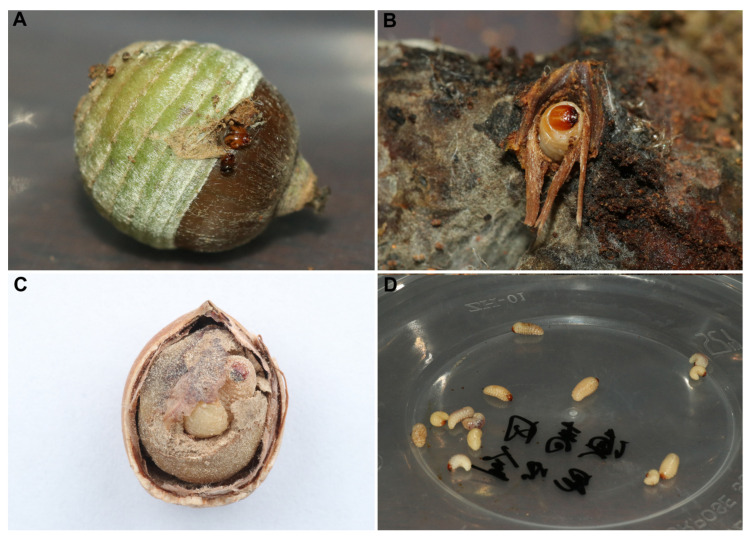
Status of weevil larvae within the acorn on the verge of boring out and those remaining inside. (**A**) Weevil larvae in *Q. schottkyana* biting through the pericarp for emergence; (**B**) weevil larvae in *L. dealbatus* emerging from the cupule at the cicatrix; (**C**) weevil larvae within *C. delavayi* that have not yet bored out; and (**D**) weevil larvae in *Q. schottkyana* that have emerged simultaneously in large numbers.

We found weevil larvae in varying numbers across all six dominant acorns in the SEBFs. However, species identification based on morphology proved challenging (Figure 2 and Figure S2).

**Figure 3:**
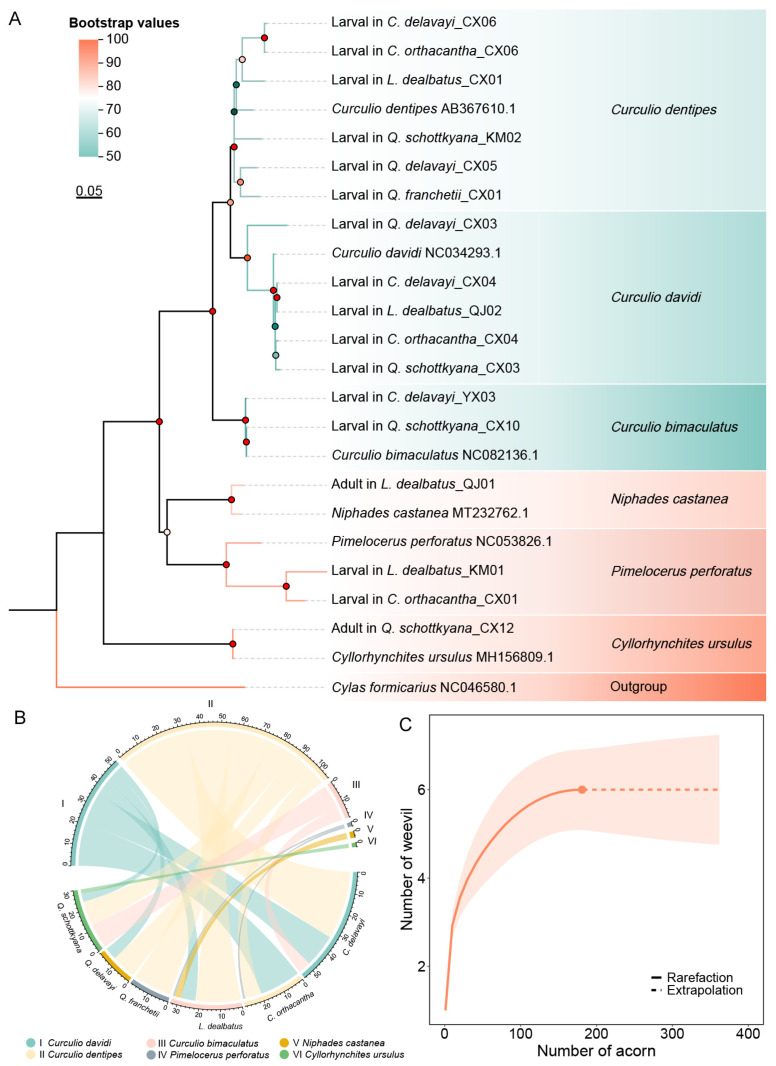
(**A**) Maximum likelihood (ML) phylogenetic tree of weevils based on *COI* DNA barcode sequences, with *Cylas formicarius* as the outgroup. Circles at nodes indicate the bootstrap values with 1000 iterations. Leaf node labels denote “larva/adult-host acorn-collection site-sample number”. Different colours denote weevil samples grouped by species. (**B**) Feeding relationships between host acorns and acorn weevils, with links indicating the presence of weevil species within specific acorns. Link width represents the number of weevils sampled per acorn species. The numbers in upper half circle indicate individual counts per weevil species; lower half numbers indicate counts per acorn species. (**C**) Species richness of acorn weevils plotted against acorn number using a sparse curve.

We conducted PCR amplification and sequencing on DNA samples from 233 acorn weevils collected from the six dominant acorns in SEBFs, resulting in 184 distinct and unambiguous *COI* sequences with no peak multiplicity. After splicing and proofreading, we obtained a 730 bp fragment sequence at the 5′-end. Phylogenetic tree construction revealed that all weevil species in SEBFs fell into six distinct genetic dendrograms, and that the test weevil sequences were matched to six species across two families and four genera. These included *Curculio dentipes* (Roelofs, 1874), *C. davidi* (Fairmaire, 1878), and *C. bimaculatus* (Marsham, 1802) from the genus *Curculio* in the family Curculionidae; *Niphades castanea* (Chao, 1980) from the genus *Niphades* Pascoe, 1871; *Pimelocerus perforatus* (Roelofs, 1873) from the genus *Pimelocerus* Roelofs, 1873; and *Cyllorhynchites ursulus* (Roelofs, 1874) from the genus *Cyllorhynchites* Voss, 1930 in the family Attelabidae (Figure 3A). The K2P genetic distance analysis among weevil species showed that the minimum inter-branch genetic distance (11.63%) was 0.5 times the maximum intra-branch genetic distance (21.15%). The average genetic distance between branches was 0.185, and the average genetic distance within branches was 6.48%. The genetic distances among the evolved branches of the three *Curculio* species ranged from 11.63% to 13.04%. *N. castanea*, *P. perforatus*, and *Cyllorhynchites ursulus* exhibited the largest genetic distances from other evolved branches, all exceeding 18%, with *C. ursulus* showing over 26% from other evolved branches (Table 2).

### 3.2. Host Range and Specificity of Weevils in SEBFs

The relationship between acorn weevils feeding on dominant acorns in SEBFs and their hosts showed considerable variation in host ranges among different acorn weevil species (Figure 3B, Table S7). Among the six acorn weevil species, *Curculio dentipes* had the highest individual count and the broadest host range, being able to consume six dominant acorn species in SEBFs. *C. davidi* feeds on five of these species, excluding *Quercus franchetii*. In contrast, *C. bimaculatus* and *P. perforatus* have more limited host ranges. *C. bimaculatus* only feeds on the two dominant acorns, *Q. schottkyana* and *Castanopsis delavayi*; *P. perforatus* only feeds on *Lithorcarpus dealbatus* and *C. orthacantha*. *Niphades castanea* and *Curculio ursulus* were the least abundant and had the most restricted host ranges. *N. castanea* only feeds on *L. dealbatus*, and *Cyllorhynchites ursulus* only on *Q. schottkyana*.

The host range and specificity analysis revealed that *N. castanea* and *Cyllorhynchites ursulus* were identified as host-specific species in this study, each exhibiting a host specificity index of one. This suggests high host specificity for both species within SEBFs. The remaining species were classified as generalists, with host specificity indices ranging from 0.14 to 0.35, with an average of 0.25, indicating low host specificity and a broad host range. Notably, *Curculio dentipes*, with the lowest host specificity index, corresponded to the broadest host range (Table 3).

### 3.3. Species Diversity of Acorn Weevils Within SEBFs

**Figure 4:**
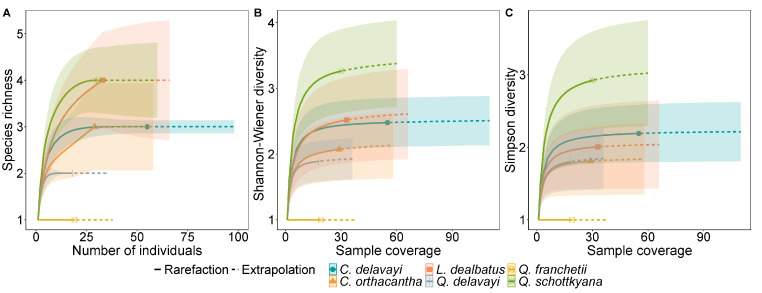
Sparse extrapolation curves show species diversity of acorn weevils in dominant acorns within SEBFs. Curves are plotted against sample number, with shaded areas indicating the 95% confidence intervals. Overlapping confidence intervals suggest no statistically significant differences in diversity indices among the species. (**A**–**C**) represent species richness, Shannon–Wiener diversity, and Simpson diversity, respectively.

The species accumulation curve of acorn weevils in relation to the sample number indicated that the rarefaction curve initially exhibited logarithmic growth and subsequently displayed a distinct inflexion point. Upon reaching six weevil species, the curve became a plateau (Figure 3C). The findings suggested that the sampling of weevil larvae for this study was sufficiently comprehensive; thus, the likelihood of encountering additional weevil species by further increasing the number of acorn weevil samples in dominant acorns of SEBFs was minimal. Analysis using the iNEXT model of acorn weevil species diversity across different host acorns revealed that all three diversity indices were consistent among host plants in SEBFs, with *Q. franchetii* showing the lowest diversity index, and no significant differences observed in acorn weevil species diversity among the other host plants (Figure 4).

### 3.4. Effects of AFTs on Acorn Weevil Species Diversity

The correlation analysis between weevil species diversity and the AFTs of host acorns did not reveal any significant correlation between the species richness of acorn weevils and the AFTs of host acorns in SEBFs. However, Shannon–Wiener diversity showed a significant negative correlation with the total flavonoid content (*R* = −0.508, *p* < 0.05) and a significant positive correlation with the starch content (*R* = 0.446, *p* < 0.05). The Simpson diversity also showed a significant negative correlation with the pericarp thickness (*R* = −0.491, *p* < 0.05) and total flavonoid content (*R* = −0.471, *p* < 0.05), as well as tannin content (*R* = −0.471, *p* < 0.05), and a significant positive correlation with the starch content (*R* = 0.476, *p* < 0.05) (Table 4).

**Figure 5:**
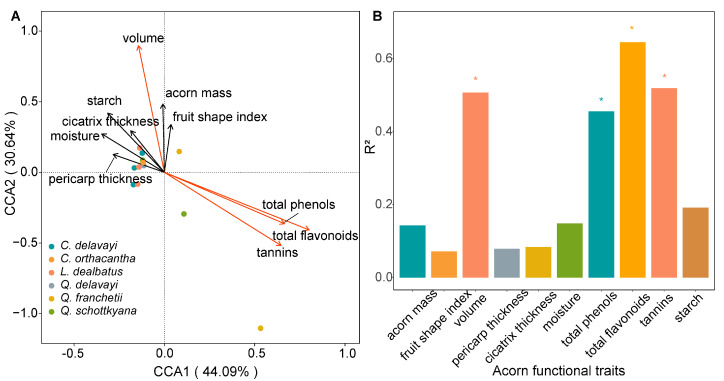
Canonical correspondence analysis (CCA) of AFTs of host acorns and species diversity of acorn weevils, including (**A**) the CCA plot and (**B**) the explanatory power of functional traits to species diversity of acorn weevils. In the CCA plot, different coloured points represent distinct host acorns, arrows from the origin indicate various acorn traits, and red arrows highlight significant trait factors in explaining weevil diversity. Traits marked with an asterisk in the bar graph indicate statistically significant differences.

Based on the outcomes of DCA and the model selection criteria (where a single-peak model is selected if the longest axis in DCA exceeds 4), CCA was employed to rank the AFTs of host acorns affecting acorn weevil species diversity. The findings revealed that the ten AFTs of host acorns explained 23.9% of the total variance in acorn weevil species diversity. The combined variance explained by the first two axes represented 74.73% of the total trait variance, capturing most of the species trait information. Thus, the first two axes were selected for two-dimensional spatial ordination. The results demonstrated that the ten AFTs of host acorns significantly influenced acorn weevil species diversity (*p* = 0.007). Furthermore, among all AFT variables, the total flavonoids in physiological traits were the most influential, followed by tannins and the total phenolic content. Among morphological traits, the acorn volume significantly affected acorn weevil species diversity in acorns from the dominant oaks of SEBFs (Figure 5, Table S8).

## 4. Discussion

### 4.1. Species Composition of Weevils in SEBFs

The near-horizontal asymptote in the species rarefaction curve of weevils suggests that the six identified weevil species are representative of the diversity of weevils feeding on dominant acorns in SEBFs. Weevils are known for their extreme diversity and widespread distribution, and while numerous studies have been conducted, precise species numbers remain elusive [7,56]. Since 1980, studies have consistently revealed the presence of *Curculio dentipes*, *C. davidi*, and *C. bimaculatus* in chestnut-producing regions across Yunnan [56,57,58,59]. Our study corroborates these findings and extends them to include *Cyllorhynchites ursulus* feeding on additional Fagaceae species prevalent in central Yunnan, such as *Quercus schottkyana* and *Q. franchetii* [31,32,60,61]. This expands upon previous observations that these species primarily feed on chestnuts in Yunnan.

Additionally, our study, supported by existing research, has found that *N. castanea* preys on acorns of the genus *Lithocarpus* Blume, 1826, and extends these observations to include *L. dealbatus*, beyond the three *Lithocarpus* species (*L. chintungensis*, *L. pachyphyllus* var. *fruticosus*, and *L. xylocarpus*) in the Ailao Mountains, located in south-central Yunnan [62]. The complex host range of *Pimelocerus perforatus* includes species from families such as Rutaceae, Meliaceae, and Oleaceae [59,63]. This is particularly noteworthy, as this study documents a new record of *P*. *perforatus* parasitizing Fagaceae acorns.

Besides the species we mentioned, other studies have found that the weevil species that feed on *Q. schottkyana* also include *Curculio megadens* [31,33,64]. However, our study did not detect this species. This may be due to a lack of DNA sequences in GenBank or its classification within the same evolutionary branch as closely related species. Increasing genomic research on *C. megadens* is necessary for fully understanding the species composition of acorn weevils [45]. Additionally, compared to the studies by Li et al. (2006) and Chen et al. (2010), which included collections from both the tree–shrub–grass layers and the soil surface layer, reported higher species counts than our study focusing solely on acorn weevils [65,66]. Understanding the composition of acorn weevils in SEBFs is essential for developing effective management strategies, as these insects significantly impact oak regeneration.

### 4.2. Host Range and Specificity of Acorn Weevils

The coexistence of multiple oak species in SEBFs provides parasitic weevils with diverse acorn resources [67,68]. However, these weevils exhibit significant differences in host range and specificity. *Curculio dentipes*, with a broad host range, consumes the acorns of all six dominant oak species in SEBFs. Outside of the Yunnan region, *C. dentipes* often feeds on *Q. mongolica*, *Q. acutissima*, *Q. variabilis*, and *Castanea mollissima*, and coexists with other weevils; *C. davidi* has been recorded on 62 species of economic plants in four families, including the *Quercus* species [58,69,70,71,72]. Its broad feeding strategy aligns with temperate weevils’ low specialization, likely adapting to seasonal host resource fluctuations or weaker interspecific competition in temperate zones [73].

*Curculio bimaculatus* was initially recorded in Yunnan and Henan, and was often mixed with *C. dentipes* in Yunnan’s chestnut plantation areas [58]. However, 13 host species for *C. bimaculatus* in Zhejiang and eastern Fujian were discovered later, except chestnut [74,75]. Our study shows that the hosts of *C. bimaculatus* also include *Q. schottkyana*, *C. delavayi*, and *L. dealbatus*, and further surveys may reveal additional oak species as hosts. This suggests that *C. bimaculatus* is also a widespread generalist insect in oak forests with low host specificity. In contrast, tropical weevils usually show stricter host specificity (e.g., Nicaraguan weevils feed on only 1–2 oak species) [73]. The subtropical weevil community in this study is still dominated by generalists, possibly reflecting transitional host specialization along the latitudinal gradient.

In addition, *P. perforatus*, found in the acorns of *L. dealbatus* and *Castanopsis orthacantha*, is widely distributed, occurring in northern and southwestern China, as well as in Japan [76,77]. It has a wide host range and low host specificity, infesting branches of multiple family species. *L. dealbatus* and *C. orthacantha*, often multi-fruited in clusters, make them high host choices [78]. *N. castanea* has only been recorded eating some species of *Castanea* and *Lithocarpus* [62,79]. It is a specialist in feeding only on *L. dealbatus* in SEBFs, with a high level of host specificity. *Cyllorhynchites ursulus* mainly feeds on chestnuts in many places, but mostly on *Q. schottkyana* in SEBFs [31,33,80]. This strict host dependence is analogous to the “niche conservatism” of tropical weevils, suggesting that cases of local specialization still exist in subtropical forests [73]. Given the limited distribution of SEBFs, the survival of such weevils highly depends on the continuity of host plants, and may also be sensitive to habitat fragmentation [81].

### 4.3. Effects of AFTs on the Species Diversity of Weevils

In terms of morphological traits, the species diversity of acorn weevils decreases with increasing pericarp thickness. This variation is attributed to differences in pericarp thickness, weevil rostrum length, bite force (the mechanical force applied by the mouthparts during feeding), and the feeding preferences among acorn species [24,25]. Additionally, acorn volume (size) significantly impacts weevil diversity. From a reproductive point of view, adult females tend to lay their eggs in larger acorns, which apparently provide more nutritional support for larval development [23,82,83]. Muñoz et al. (2014) found that acorn size influences the body size of acorn weevils in the Mediterranean region. The weevils exhibited a degree of specificity between hosts, which was primarily driven by acorn size [2]. Consequently, to access more favourable nutrition, acorn weevils may prefer larger acorns with thinner pericarps during feeding and reproduction, potentially attracting a greater diversity of weevil species [73,84].

Acorn chemical defences significantly influenced the species diversity of acorn weevils [26]. Our study reveals a notable negative correlation between weevil diversity and the chemical defences of their host acorns. Early research indicates that some phytophagous insects secrete specific defence elicitors, such as fatty acid conjugates (FACs), which can provoke a defensive response in plants [85]. The egg-laying behaviour of parasitic insects can also trigger host plant defences. For example, when *Callosobruchus maculatus* and *Bruchus pisorum* lay their eggs, the host peas recognize signalling material on the eggs and form pod tissues, preventing newly hatched weevil larvae from entering the pods [86]. Furthermore, some plants synthesize tannins in response to insect damage, which limits the synthesis of midgut proteins and digestive enzymes, ultimately restricting insect growth and development [29,87]. Consequently, high chemical defences in acorns may reduce their palatability, decreasing feeding by acorn weevils and, in turn, their species diversity.

Insects have developed various adaptations to counter plant defences, and the most common is resistance to secondary metabolites [88]. For example, *Salvia ceratophylloides* Ard. produces essential oils, yet does not effectively resist the parasitism of *Squamapion elongatum* (Germar,1817) (Coleoptera, Curculionoidea, and Apionidae), which may be related to *S. elongatum*’s counteradaptation to the defensive mechanisms of host plant [8]. The presence of *Serratia marcescens* and *Lactococcus lactis* with tannase activity in the gut flora of *Curculio dentipes* aids in the uptake of high-tannin foods, potentially explaining the ability of some weevil larvae to adapt to high-tannin diets [89]. Generally, acorns with high levels of secondary metabolites deter feeding by less defended weevils, reducing acorn weevil species diversity. Acorn weevils have evolved defensive mechanisms to better cope with these defences, enhancing their survival chances. This continuous interplay of defence, evasion, and co-adaptation between the species in an ‘arms race’ for survival enriches and expands the diversity of their populations.

## 5. Conclusions

In the semi-humid evergreen broad-leaved forests of southwest China, six acorn weevil species across four genera and two families were identified with the aid of molecular techniques. These included *Curculio dentipes*, *C. davidi*, *C. bimaculatus*, *Niphades castanea*, and *Pimelocerus perforatus* of the family Curculionidae, and *Cyllorhynchites ursulus* of the family Attelabidae. These species exhibited varying degrees of host specificity, with some showing broad host ranges and others being highly host specific. Notably, no significant differences in the species diversity of acorn weevils were observed among the dominant acorns, except for *Quercus franchetii*. However, acorn functional traits significantly influenced the species diversity of acorn weevils, including host acorn volume and secondary metabolite contents such as total phenols, total flavonoids, and tannins. Our results provide a foundation for further research into the mechanisms driving these interactions and their broader ecological implications, which are crucial for the ecological management and biodiversity conservation of similar forests.

## Figures and Tables

**Table 1:** Sampling information of weevils used for molecular work.

Host Species	Locality	No. of Weevils	Total
*Castanopsis delavayi*	Xishan, Kunming	11	57
	Huaning, Yuxi	3	
	Yao’an, Chuxiong	43	
*C*. *orthacantha*	Xinping, Yuxi	12	42
	Zixi, Chuxiong	5	
	Yao’an, Chuxiong	25	
*Lithocarpus dealbatus*	Luoping, Qujing	13 (2) *	36
	Xishan, Kunming	4	
	Lufeng, Chuxiong	17	
*Quercus delavayi*	Luquan, Kunming	11	40
	Luquan, Kunming	13	
	Yao’an, Chuxiong	16	
*Q*. *franchetii*	Binchuan, Dali	15	22
	E’shan, Yuxi	5	
	Xishan, Kunming	2	
*Q*. *schottkyana*	Xishan, Kunming	12 (2) *	36
	Yao’an, Chuxiong	13	
	Yongren, Chuxiong	11	

Note: The column of “locality” indicates “county/township, municipality”; * number of adult weevils in parentheses.

**Table 2:** Kimura two-parameter (K2P) distances (in percentages) between all acorn weevil taxa calculated from the DNA barcode sequences (*COI*), with species identified as in the ML phylogenetic tree in Figure 3A.

	1	2	3	4	5	6
1. *Curculio dentipes*	8.50					
2. *C*. *davidi*	11.63	6.70				
3. *C*. *bimaculatus*	12.18	13.04	2.83			
4. *Niphades castanea*	20.39	19.85	18.40	4.38		
5. *Pimelocerus perforatus*	21.96	21.60	20.91	20.12	15.03	
6. *Cyllorhynchites ursulus*	26.85	27.22	27.19	26.23	29.74	1.41

**Table 3:** The host range and specificity of weevil species feeding on dominant acorns in SEBFs.

Species of Weevil		No. of Host Species	No. of Host Genus	Host Specificity (*S*)
Generalist species	*Curculio davidi*	5.00	3.00	0.15
	*C. dentipes*	6.00	3.00	0.14
	*C. bimaculatus*	2.00	2.00	0.35
	*Pimelocerus perforatus*	2.00	2.00	0.35
	Average	3.75	2.50	0.25
Host-specific species	*Niphades castanea*	1.00	1.00	1.00
	*Cyllorhynchites ursulus*	1.00	1.00	1.00
	Average	1.00	1.00	1.00

**Table 4:** Correlation between acorn weevil species diversity and AFTs of host acorn within SEBFs.

Trait Variable		Species Richness	Shannon–Wiener Diversity	Simpson Diversity
Morphological traits	Acorn mass	−0.05	−0.15	−0.23
	Fruit shape index	0.35	0.35	0.30
	Volume	0.08	0.18	0.05
	Pericarp thickness	−0.38	−0.32	−0.49 *
	Cicatrix thickness	0.18	0.14	0.04
Physiological traits	Moisture	0.13	−0.04	0.04
	Total phenols	−0.41	−0.20	−0.43
	Total flavonoids	−0.42	−0.51 *	−0.47 *
	Tannins	−0.40	−0.41	−0.47 *
	Starch	0.37	0.45 *	0.48 *

Note: * *p* < 0.05, the difference is significant.

## Data Availability

The original contributions presented in this study are included in the article/supplementary material. Further inquiries can be directed to the corresponding author.

## Supplementary Materials

The following supporting information can be downloaded at: https://www.mdpi.com/article/10.3390/insects16060579/s1. Table S1: acorn sampling data and sample counts from dominant host oaks in SEBFs; Table S2: acorn functional traits and their ecological roles in oak species; Table S3: the volume fitting formula for acorns of different oak species; Table S4: detailed taxonomic resources used in present study; Table S5: optimal nucleotide substitution model for maximum likelihood phylogenetic trees; Table S6: functional trait values of acorns from dominant oak species in SEBFs; Table S7: the number of acorns consumed by acorn weevils of different species; Table S8: correlation coefficient and significance level between AFTs of host and acorn weevil species diversity; Figure S1: examples of morphometric measurements of acorns; Figure S2: morphology of acorns from dominant oak species and their state following weevil parasitism in SEBFs.
