# Supplementary material for: Acorn Weevil Species Diversity and Host Affinity in the Semi-Humid Evergreen Broad-Leaved Forests of Southwest China
Source: Insects. 2025 May 30;16(6):579. doi: 10.3390/insects16060579 (PMC12193683; doi:10.3390/insects16060579)
Supplement: Supplementary file 1 [file insects-16-00579-s001.zip › insects-3608309-supplementary.pdf]

insects

MDPI

Supplementary Materials

Acorn Weevil Species Diversity and Host Affinity in the Semi-Humid Evergreen Broad-Leaved Forests of Southwest China

Shengquan Fang [1,2,3], Shaoji Hu [4,5], Biao Zhao [1,2], Dengpeng Chen [1,2], Chunyan Lan [1,2], Xinrong Li [1,2], Yongping Li [6], Mingchun Peng [1,2], Zihao Wang [1,2], Mingyu Ge [1,2] and Chongyun Wang [1,2,*]

School of Ecology and Environmental Science, Yunnan University, Kunming 650500, China; shengquanfang@hotmail.com (S.-Q.F.); 18213535564@163.com (B.Z.); chendengpeng@itc.ynu.edu.cn (D.-P.C.); lcy_61208@126.com (C.-Y.L.); x571142369@163.com (X.-R.L.); mchpeng@ynu.edu.cn (M.-C.P.); w15675011759@163.com (Z.-H.W.); 17387415438@163.com (M.-Y.G.)
[2] Institute of Ecology and Geobotany, Yunnan University, Kunming 650500, China
[3] Yuxi Institute of Ecology and Environment Science, Yuxi 653100, China
[4] Institute of International Rivers and Eco-Security, Yunnan University, Kunming 650500, China; shaojihu@hotmail.com
[5] Yunnan Key Laboratory of International Rivers and Transboundary Eco-Security, Yunnan University, Kunming 650500, China
[6] School of Agriculture, Yunnan University, Kunming 650500, China; liyp@ynu.edu.cn
* Correspondence: cywang@ynu.edu.cn

Table S1. Acorn sampling data and sample counts from dominant host oaks in SEBFs.

Oak species	Longitude	Latitude	Locality	Collection date	Number of acorns	Total
	101.054778	25.616378	Yaoan, Chuxiong	2023-X	215	
Castanopsis delavayi	102.629021	24.969602	Xishan, Kunming	2022-X	409	868
	102.882753	24.235641	Huaning, Yuxi	2022-XI	244	
	101.087907	25.617267	Yaoan, Chuxiong	2023-X	125	
Castanopsis orthacantha	101.406044	25.024057	Chuxiong,Chuxiong	2022-X	72	429
	101.940652	23.972165	Xinping, Yuxi	2022-X	232	
	101.806269	25.193116	Lufeng, Chuxiong	2023-X	320	
Lithocarpus dealbatus	102.575142	24.840145	Xishan, Kunming	2022-X	88	908
	104.183118	25.099587	Luoping, Qujing	2022-X	500	
	101.093859	25.61401	Yaoan, Chuxiong	2023-X	312	
Quercus delavayi	102.379057	25.727054	Luquan, Kunming	2022-X	575	1173
	102.691412	25.680281	Luquan, Kunming	2022-XI	286	
	101.802796	26.158986	Yongren, Chuxiong	2022-XI	100	
Quercus franchetii	102.39629	24.160946	Eshan, Yuxi	2022-XI	331	753
	102.604178	24.846233	Xishan, Kunming	2022-X	322	
	101.291904	25.466706	Yaoan, Chuxiong	2022-X	719	
Quercus schottkyana	101.799374	26.159017	Yongren, Chuxiong	2022-X	613	1817
	102.629153	24.962467	Xishan, Kunming	2022-XI	485	

Table S2. Acorn functional traits and their ecological roles in oak species.

Functional traits	Traits	Acronym	Functions	Unit
	acorn mass	AM	Resource capture	g
	Fruit shape index	FSI	Resource capture	-
morphological traits	Volume	VO	Resource capture	cm^3
	Pericarp thickness	PT	Structure and defence	mm
	Cicatrix thickness	CIT	Structure and defence	mm

Physiological traits	Moisture content	MC	Resource capture	%
	Total phenols	TP	Resource capture and defence	mg/g
	Total flavonoids	TF	Resource capture and defence	mg/g
	Tannins	TA	Resource capture and defence	mg/g
	Starch	ST	Resource capture	mg/g

Table S3. The volume fitting formula for acorns of different oak species.

Host plant	The fitting formula of volume	R^2
Quercus franchetii	V=0.101*L-0.024*T	0.929
Quercus schottkyana	V=-0.029*L+0.125*T	0.989
Quercus delavayi	V=-0.115*L+0.259*T	0.983
Lithocarpus dealbatus	V=0.014*L+0.073*T	0.989
Castanopsis delavayi	V=-0.015*L+0.091*T	0.959
Castanopsis orthacantha	V=-0.021*L+0.137*T	0.960

Note: V is acorn volume, L is longitudinal diameter, and T is transverse diameter.

Table S4. Detailed taxonomic resources used in present study.

Family	Genus	Species	GenBank access
Curculionidae	*Curculio*	*Curculio dentipes* Roelofs , 1874	AB367610
		Curculio davidi Fairmaire, 1878	NC034293
		Curculio bimaculatus Marsham, 1802	NC082136
	Niphades	*Niphades castanea* Chao, 1980	MT232762
	Pimelocerus	*Pimelocerus perforatus* Roelofs, 1873	NC053826
Attelabidae	*Cyllorhynchites*	*Cyllorhynchites ursulus* Roelofs , 1874	MH156809
Brentidae	*Cylas*	*Cylas formicarius* Fabricius, 1793	NC046580

Table S5. Optimal nucleotide substitution model for maximum likelihood phylogenetic trees.

Model	#Param	BIC	AICc	lnL	Invariant	Gamma	R
GTR+G+I	55	9914.08	9488.56	-4689.10	0.46	1.13	2.95
TN93+G+I	52	9941.87	9539.54	-4717.61	0.46	0.99	3.07
GTR+G	54	9947.62	9529.83	-4710.74	n/a	0.27	2.41
TN93+G	51	9960.01	9565.41	-4731.55	n/a	0.25	2.85
GTR+I	54	9995.25	9577.46	-4734.55	0.53	n/a	2.21
HKY+G+I	51	10008.79	9614.20	-4755.94	0.46	1.00	2.54
T92+G+I	49	10012.64	9633.50	-4767.61	0.47	1.01	2.65
HKY+G	50	10015.60	9628.74	-4764.22	n/a	0.24	2.57
T92+G	48	10020.35	9648.94	-4776.33	n/a	0.24	2.68
TN93+I	51	10057.73	9663.13	-4780.41	0.53	n/a	1.50

Table S6. Functional trait values of acorns from dominant oak species in SEBFs.

Acorn species	*Castanopsis delavayi*	*Castanopsis orthacantha*	*Lithocarpus dealbatus*	*Quercus delavayi*	*Quercus franchetii*	*Quercus schottkyana*
			**morphological traits**			
acorn mass	0.61±0.25	0.53±0.06	0.76±0.2	1.56±0.21	0.4±0.07	0.74±0.29
fruit shape index	1.12±0.11	1.26±0.22	1.04±0.08	1.17±0.08	1.05±0.1	1.26±0.09
volume	0.93±0.18	1.19±0.07	1.1±0.16	1.72±0.09	0.5±0.11	0.99±0.14
pericarp thickness	0.51±0.07	0.44±0.03	0.79±0.13	0.48±0.04	0.45±0.01	0.39±0.05
cicatrix thickness	0.66±0.06	0.73±0.1	0.85±0.02	0.88±0.02	0.66±0.11	1.04±0.18

			**physiological traits**			
moisture	17.14±2.77	23.61±4.01	25.47±11.56	29.19±13.32	14.95±4.37	18.69±11.05
total phenols	15.93±2.01	16.94±2.03	23.45±3.15	42.25±15.53	133.64±15.7	125.93±6.96
total flavonoids	15.44±3.85	18.6±1.35	24.83±1.8	43.59±9.7	71.86±7.87	34.14±3.57
tannins	2.45±0.24	2.98±0.29	9.64±1.66	14.53±3.31	26.62±5.33	16.92±2.82
starch	378.19±55.81	327.35±22.47	225.17±90.98	419.01±51.3	209.16±15.7	303.43±9.64

Note: The data in the table represents: mean ± SE.

Table S7. The number of acorns consumed by acorn weevils of different species.

	Castanopsis delavayi	*Castanopsis orthacantha*	*Lithocarpus dealbatus*	*Quercus franchetii*	*Quercus delavayi*	*Quercus schottkyana*
Curculio davidi	16	20	7	0	6	4
Curculio dentipes	33	8	22	19	12	12
Curculio bimaculatus	6	0	0	0	0	12
Pimelocerus perforatus	0	1	1	0	0	0
Niphades castanea	0	0	3	0	0	0
Cyllorhynchites ursulus	0	0	0	0	0	2

Table S8. Correlation coefficient and significance level between AFTs of host and parasitic weevil species diversity.

Functional traits	CCA1	CCA2	R^2	*P.adj*
acorn mass	-0.008	0.483	0.142	0.263
fruit shape index	0.037	0.338	0.070	0.451
volume	-0.143	0.897	**0.506**	0.003
pericarp thickness	-0.284	0.127	0.078	0.440
cicatrix thickness	-0.186	0.295	0.082	0.440
moisture	-0.347	0.273	0.147	0.255
total phenols	0.665	-0.364	**0.454**	0.003
total flavonoids	0.802	-0.408	**0.644**	0.003
tannins	0.646	-0.519	**0.518**	0.005
starch	-0.313	0.419	0.190	0.234

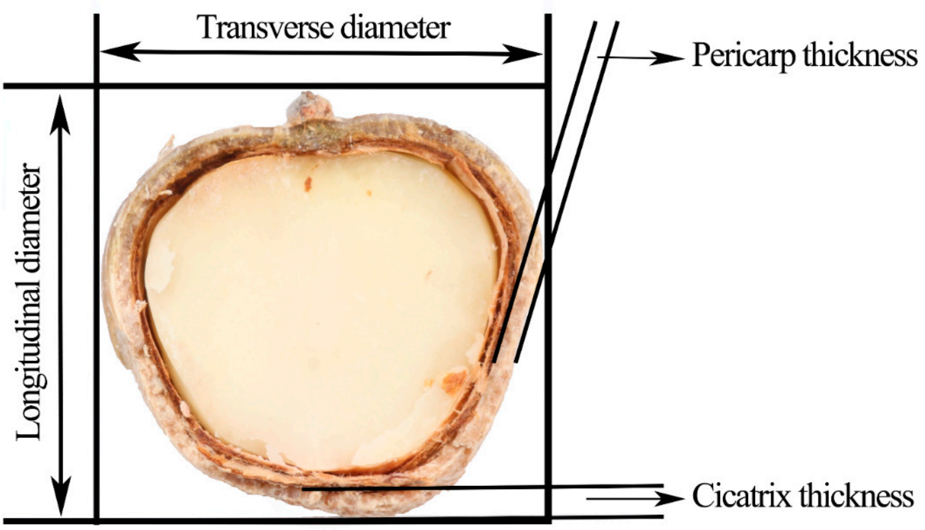

Figure S1. Examples of morphometric measurements of acorns. Examples in the figure include: Pericarp thickness, Seed coat thickness, Cicatrix thickness, Longitudinal diameter, Transverse diameter.

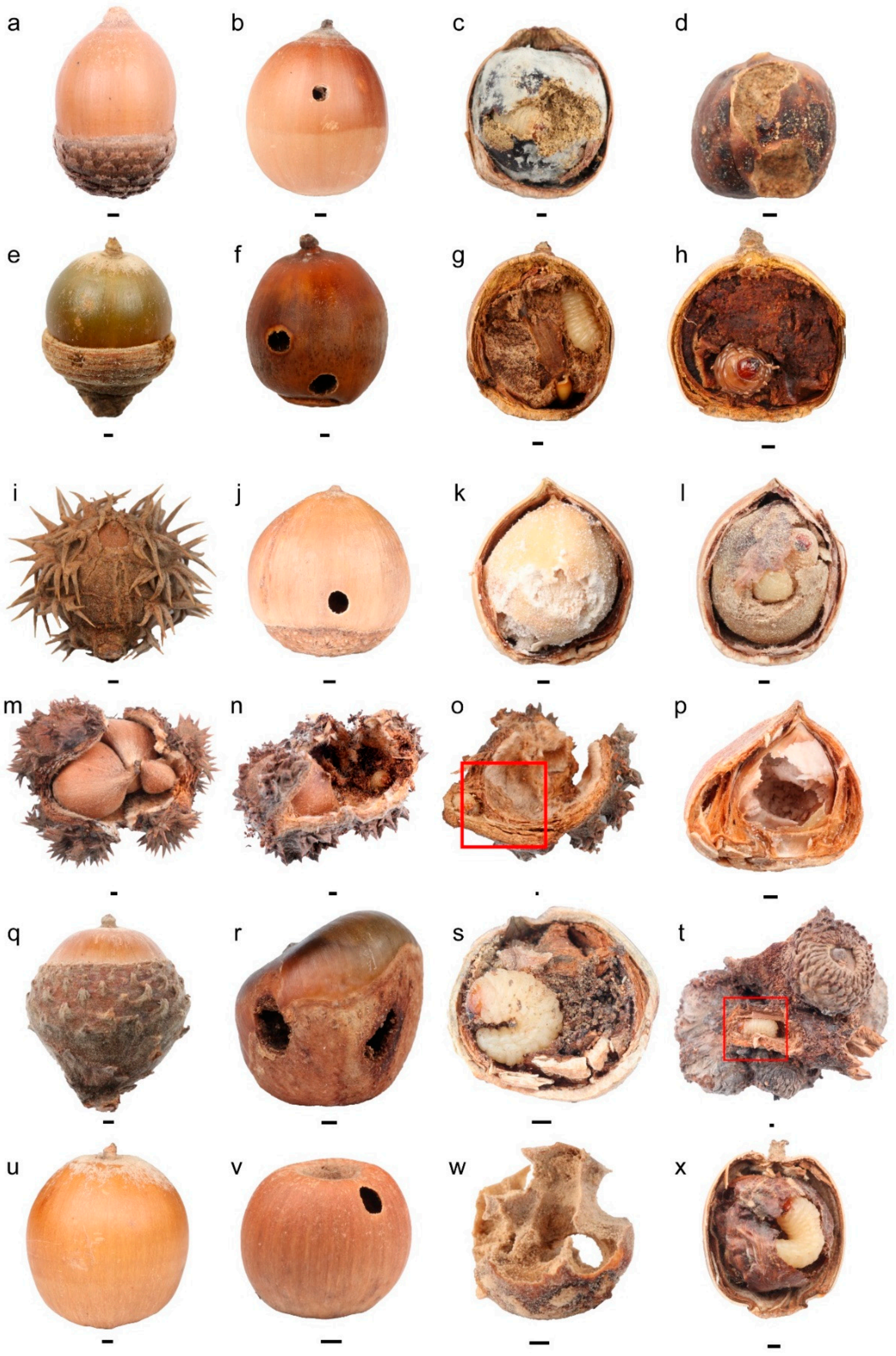

Figure S2. Morphology of acorns from dominant oak species and their state following weevil parasitism in SEBFs. a - d, *Q. schottkyana*; e - h, *Q. delavayi*; i - l, *C. delavayi*; m - p, *C. orthacantha*; q - t, *L. dealbatus*; u - x, *Q. franchetii*. The length of the scale represents 1mm.
